# hsa_circ_0000745 promotes cervical cancer by increasing cell proliferation, migration, and invasion

**DOI:** 10.1002/jcp.29045

**Published:** 2019-06-29

**Authors:** Jun Jiao, Teng Zhang, Xinlin Jiao, Tingting Huang, Lu Zhao, Daoxin Ma, Baoxia Cui

**Affiliations:** ^1^ Department of Obstetrics and Gynecology Qilu Hospital of Shandong University Jinan China; ^2^ Hematology Oncology Center Qilu Hospital of Shandong University Jinan China; ^3^ Department of Obstetrics and Gynecology Taian City Central Hospital Taian China

**Keywords:** cervical cancer, circular RNA, E‐cadherin., hsa_circ_0000745, squamous cell carcinoma

## Abstract

Circular RNAs (circRNAs) participate in gene regulation and malignant tumor progression, including uterine cervical cancer (CC). In this study, the expression profile of circRNAs in CC was detected using circRNA microarrays. Then, we selected hsa_circ_0000745 for further examination from the significantly dysregulated circRNAs. Proliferation assays, Transwell assays, quantitative reverse transcription polymerase chain reaction, western blot analysis and tumorigenesis tests in vivo were used to validate the role of hsa_circ_0000745 in CC. hsa_circ_0000745 was upregulated in CC, and its level positively correlated with the level of its linear messenger RNA isoform. Patients with poorly differentiated tumors or vascular/lymphatic invasion presented higher expression of hsa_circ_0000745. The role of hsa_circ_0000745 was illuminated by knocking down hsa_circ_0000745 in CC cells, and the results revealed that reducing hsa_circ_0000745 inhibited cell proliferation, migration, and invasion in CC by upregulating E‐cadherin (E‐cad) expression. In summary, as a tumor promoter in CC, hsa_circ_0000745 enhances the cell's ability to proliferate, migrate, and invade by reducing the expression of E‐cad. hsa_circ_0000745 is a candidate target for the treatment of CC in the clinic.

## Graphical abstract

Hsa_circ_0000745 is upregulated and functions as a tumor promoter in cervical cancer. Hsa_circ_0000745 enhances the cell's ability to proliferate, migrate, and invade by reducing the expression of E‐cadherin in cervical cancer.

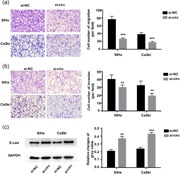

## 1 INTRODUCTION

Uterine cervical cancer (CC) ranks as the 3rd most common cancer and the 4th most common cause of cancer‐related death in women worldwide (Ferlay et al., 2015). Approximately 80% of CC cases are squamous cell carcinoma (SCC). Persistent infection with high‐risk human papillomavirus (HPV) is a primary factor but not a necessary condition that causes CC (Munoz, Castellsague, de Gonzalez, & Gissmann, 2006). HPV vaccination and early detection programs can reduce the morbidity of CC; however, the effect of these regimens is limited worldwide. Many countries with inadequate healthcare systems suffer from higher CC morbidity and mortality rates. Although its incidence ranks only 11th in developed countries, the incidence of CC ranks second in developing areas (Ginsburg et al., 2017). The mechanisms underlying the occurrence and development of CC must be elucidated to reduce the morbidity and mortality of CC in addition to high‐risk HPV infections.

Circular RNA (circRNA) belongs to noncoding RNA (ncRNA) that participates in regulating gene expression and tumor progression (Meng et al., 2017; Wang, Nazarali, & Ji, 2016). The joining of 3′ end and 5′ end of the RNA creates the circular shape of the circRNAs (Suzuki & Tsukahara, 2014). There are several mechanisms by which circRNAs participate in cellular biology. circRNAs contain multiple binding sites for microRNAs (miRNAs). circRNAs bind miRNAs at their miRNA response elements to inhibit the activities of the miRNAs and regulate their targets (Cai et al., 2019; Han et al., 2017; Hansen, Jensen, et al., 2013). Research on this role is a hotspot of circRNA research. circRNAs directly regulate the transcription of linear RNAs through competitive splicing and regulate the expression of proteins by isolating RNA‐binding proteins that normally make up RNA‐protein complexes to control the expression of genes (Li et al., 2015; Wilusz & Sharp, 2013).

The abnormal expression of circRNAs is closely related to various cancers. circZKSCAN1, which may be a useful diagnostic marker, suppresses the growth and metastasis of hepatocellular carcinoma (Yao et al., 2017). circPSMC3 inhibits the development and metastasis of gastric cancer (GC) by inhibiting functions of miR‐296‐5p (Rong et al., 2019). Aberrantly expressed circRNAs have also been identified in CC. The expression profiles of ncRNAs, including circRNA, have been explored in CC using high‐throughput RNA sequencing, and circRNAs represent potentially new diagnostic and therapeutic markers of CC (Wang, Zhao, Chen, & Cui, 2017). circ_0067934 participates in CC development by regulating miR‐545 expression and promotes the progression of CC (Hu et al., 2019). A previous study (Yu et al., 2018) identified circRNAs that are dysregulated in the response to radiation in CC cells using high‐throughput sequencing. Based on the results, these circRNAs may function in the response of CC to radiation in humans. But the specific role of circRNAs in CC still remains largely unknown.

In this study, a circRNA microarray analysis was conducted to detect the aberrantly expressed circRNAs in CC tissues. hsa_circ_0000745 (hereafter referred to as circ‐0745) was selected for in‐depth study among the dysregulated circRNAs because of its significantly abnormal expression according to the microarray result. circ‐0745 is transcribed from the sperm antigen with calponin homology and coiled‐coil domains 1 (*SPECC1*) located on chromosome 17. circ‐0745 expression was markedly upregulated in CC. Functionally, downregulation of circ‐0745 obviously suppressed the proliferation, migration, and invasion of CC cells. According to our findings, circ‐0745 promoted CC cell proliferation and metastasis; therefore, circ‐0745 represents a potential prognostic and therapeutic marker for CC patients.

## 2 MATERIALS AND METHODS

### 2.1 Specimen collection

CC tissues and adjacent nontumorous cervical tissues were obtained from patients at Qilu Hospital of Shandong University, Jinan, China. These patients’ preoperative pathologic examination showed cervical SCC. All samples were macrodissected within 15 min after uterine resection, and the postoperative pathological analysis confirmed that the specimens were SCC. The ethical approval for the study was provided by the Medical Ethics Committee of Qilu Hospital. All patients provided written informed consent. The clinical stages of the patients with SCC were determined according to the International Federation of Gynecology and Obstetrics (FIGO) criteria.

### 2.2 Cell culture

SiHa and CaSki cells were from the gynecological laboratory at Qilu Hospital, China. SiHa and CaSki cells were cultured in α‐minimum essential medium (Gibco‐BRL, Grand Island) and Roswell Park Memorial Institute 1640 (Gibco‐BRL), adding 10% fetal bovine serum (Gibco‐BRL), respectively.

### 2.3 circRNA microarray analysis

All samples were ground into powder and RNAs were extracted with TRIzol (Invitrogen, Carlsbad). The purity and quantity of the RNAs in the specimens were evaluated using a NanoDrop ND‐1000 (Implen, Munich, Germany).

We selected five pairs of SCC tissues and paracancerous tissues from patients. The circRNA microarray (Arraystar Inc., Rockville) was conducted following the manufacturer's protocol. The detected circRNAs in two groups were analyzed by the false discovery rate and paired *t* tests. Significantly differentially expressed circRNAs were defined as circRNAs with a fold change >2.0 and a *p* value <.05. The clinicopathological data from the patients are shown in Table 1.

**Table 1:** Clinical and histopathological characteristics of SCC patients for circRNA microarray analysis

Patient ID	Age	Clinical stage	Differentiation	Tumor size (cm)	Lymphnode metastases	Vascular/lymphatic invasion
1	32	IB2	Moderate	≥4	Negative	Positive
2	32	IB1	Low	<4	Negative	Negative
3	41	IB1	Low	<4	Negative	Negative
4	44	IIA	Low	<4	Negative	Negative
5	41	IB1	High	<4	Negative	Negative

Abbreviation: circRNA, circular RNA; SCC, squamous cell carcinoma.

### 2.4 Quantitative reverse transcription polymerase chain reaction (qRT‐PCR) analysis

RNAs from 59 paired samples were utilized to measure the expression of circ‐0745 and the linear *SPECC1* messenger RNA (mRNA) using qRT‐PCR analysis. SuperScript III RT (Invitrogen) was used to synthesize complementary DNA from 3 µg of total RNA. The PCR mixture contained 2 µl of the complementary DNA templates, a 1 × final concentration of SYBR Green (Toyobo, Osaka, Japan), and the specific primers. The sequences of the primers synthesized by Biosune (Shanghai, China) are shown in Table 2. All tests were conducted in triplicate on a LightCycler 480 II (Roche, Basel, Switzerland). Relative expressions of circ‐0745 and linear *SPECC1* mRNA were calculated with $2^{{-\Delta \Delta C}_{t}}$ method.

**Table 2:** The sequences of primers for qRT‐PCR and siRNAs

RNA	Sequence
hsa_GAPDH forward sequence	5′‐GGGAAACTGTGGCGTGAT‐3′
hsa_GAPDH reverse sequence	5′‐GAGTGGGTGTCGCTGTTGA‐3′
hsa_circ_0000745 forward sequence	5′‐ATGTTGAAAGTAGCCCGAGCAG‐3′
hsa_circ_0000745 reverse sequence	5′‐TGGGAGTGTTGGAAGAAGTTGG‐3′
Linear *SPECC1* mRNA forward sequence	5′‐ACCCCAGGAAATCAGTGTCCA‐3′
Linear *SPECC1* mRNA reverse sequence	5′‐GTTCCCGAACTTGGGACTCAA‐3′
siRNA‐1	5′‐CTGGCCAAGGGGCCUUUACTT‐3′
siRNA‐2	5′‐CUGGCCAAGGGGCCUUUACATT‐3′
siRNA‐3	5′‐GUCUGCUGGCCAAGGGGCCTT‐3′
si‐Negative control	5′‐UUCUUCGAACGUGUCACGUTT‐3′

*Note*: GAPDH, glyceraldehyde 3‐phosphate dehydrogenase; mRNA, messenger RNA; qRT‐PCR, quantitative reverse transcription polymerase chain reaction; siRNA, small interfering RNA.

### 2.5 Silencing of circ‐0745 in CC cells

Three small interfering RNAs (siRNAs) and a negative siRNA control (si‐NC) were synthesized (GenePharma, Shanghai, China). The siRNAs targeted the junction site of circ‐0745 and the sequences of siRNAs are shown in Table 2. SiRNAs were transfected into CaSki and SiHa cells with Lipofectamine 2000 (Invitrogen). The interference efficiency was measured using qRT‐PCR after 48 hr. The most effective siRNA was selected for all subsequent experiments.

### 2.6 Cell viability assay and apoptosis assay

After transfection with siRNAs, cells were seeded and viability was measured after culture for 0, 24, 48, and 72 hr by adding Cell Counting Kit‐8 reagents (CCK‐8; Solarbio, Beijing, China) and incubating at 37°C for 2 hr. All tests were conducted at least three times. The absorbance of the cells at 450 nm was detected using a Model 680 Microplate Reader (Bio‐Rad, Hercules).

The transfected cells were collected and labeled with Annexin V‐FITC (Sigma, St. Louis) and propidium iodide (Sigma). The respective fluorescence intensities were detected on a Beckman Gallios flow cytometer (Beckman, Brea).

### 2.7 Cell migration and invasion assay

Transwell chambers (Corning) without and with a basement membrane coating (Corning) were used to detect the mobility of transfected CC cells, respectively. For migration test, 2.5 × 10^4^ cells were placed into chambers of Transwell inserts without basement membrane coating; for the invasion test, 5 × 10^4^ cells were seeded in chambers of Transwell inserts with a basement membrane coating. After 24 hr, nonmigratory or noninvasive cells were wiped and the cells migrating through the membrane were counted under a microscope (Olympus, Tokyo, Japan).

### 2.8 Subcutaneous tumor formation in mice

The sequence of siRNA‐2 was inserted a lentiviral vector for the stable knocked down of circ‐0745 (named sh‐circ‐0000745). sh‐circ‐0000745 and an empty lentiviral vector as nonsense control (Genechem Co., Shanghai, China) were constructed containing the green fluorescent protein gene and a puromycin resistance gene. CaSki cells were transfected and selected with puromycin (2 μg/ml; Sigma) to obtain stable cell clones.

All animal care and experimental procedures were conducted according to the standards of the National Institutes of Health and approved by the Animal Protection and Use Committee of Shandong University. First, 10^7^ stably transfected CaSki cells were subcutaneously injected into the backs of female BALB/c mice (5 weeks old). The tumors were monitored weekly. The mice were killed after 4 weeks, and tumors were excised and assessed.

### 2.9 Western blot analysis

After transfected for 48 hr, CaSki and SiHa cells were lysed with radioimmunoprecipitation assay buffer (Sigma). Proteins were extracted and electrophoresed on 10% sodium dodecyl sulfate‐polyacrylamide gels, then transferred to nitrocellulose filter membranes. The membranes were incubated overnight with a primary E‐cadherin antibody (E‐cad, 1:1000; Cell Signaling Technology, Boston) and glyceraldehyde 3‐phosphate dehydrogenase (GAPDH) antibody (1:1000; Cell Signaling Technology). Then a horseradish peroxidase‐conjugated secondary antibody (1:1000; Cell Signaling Technology) was used. Protein bands were visualized using a FluroChem E system (Protein Simple, Silicon Valley) and the signals were quantified. GAPDH was measured as a loading control.

### 2.10 Statistical analyses

Data are presented as the means ± *SD*. Comparisons between two groups were performed using Student's *t* test. Correlation analysis was performed with Pearson's analysis. Differences were considered significant at *p* < .05. All statistical analyses were performed with SPSS 18.0.

## 3 RESULTS

### 3.1 Differentially expressed circRNAs in SCC

**Figure 1:**
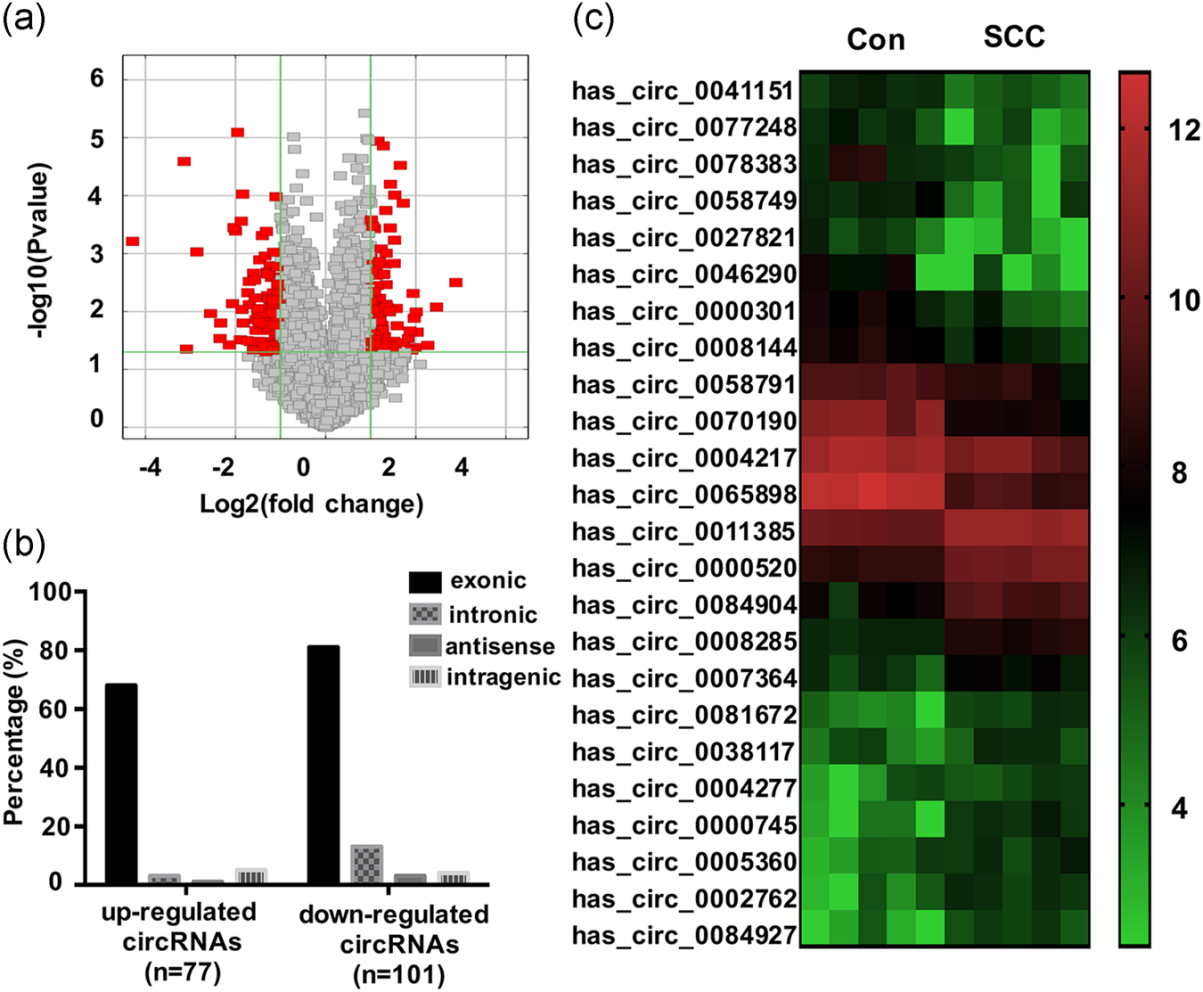
Expression profiles of circRNAs in SCC and paired adjacent normal cervical tissues. (a) In the volcano plots, the red points represent circRNAs with a >2‐fold increase or decrease in their expression (*p* < .05). The horizontal green line represents a value of *p* = .05, and the vertical green lines indicate the thresholds for a 2‐fold increase or decrease. (b) The classification percentage analysis of upregulated and downregulated circRNAs in SCC. (c) The heat map shows the expression of 12 upregulated and 12 downregulated circRNAs in SCC compared with the control tissues on a scale from low (green) to high (red). circRNA, circular RNA; SCC, squamous cell carcinoma [Color figure can be viewed at wileyonlinelibrary.com]

Overall, 3,008 human circRNAs were detected in SCC tissue and normal cervical tissue cells. One hundred seventy‐eight circRNAs exhibited abnormal expression in SCC samples (fold change > 2.0), indicating their dysregulation. Volcano plot filtering showed the expression patterns of circRNA among the samples (Figure 1a).

Further analysis found that there were 77 upregulated and 101 downregulated circRNAs in SCC tissues compared with the levels in the paired control group. Classification of the 77 upregulated circRNAs revealed 68 exonic, 3 intronic, 1 antisense, and 5 intragenic circRNAs (Figure 1b), the 101 downregulated circRNAs included 81 exonic, 13 intronic, 3 antisense, and 4 intragenic circRNAs (Figure 1b). Several circRNAs showed approximately 10‐fold changes in their expression between the normal and cancerous cervical tissues. Twenty‐four differentially expressed circRNAs are shown in Figure 1c. The microarray data has been uploaded to the NCBI Gene Expression Omnibus (GEO) website (GSE102686).

### 3.2 circ‐0745 is upregulated in SCC

**Figure 2:**
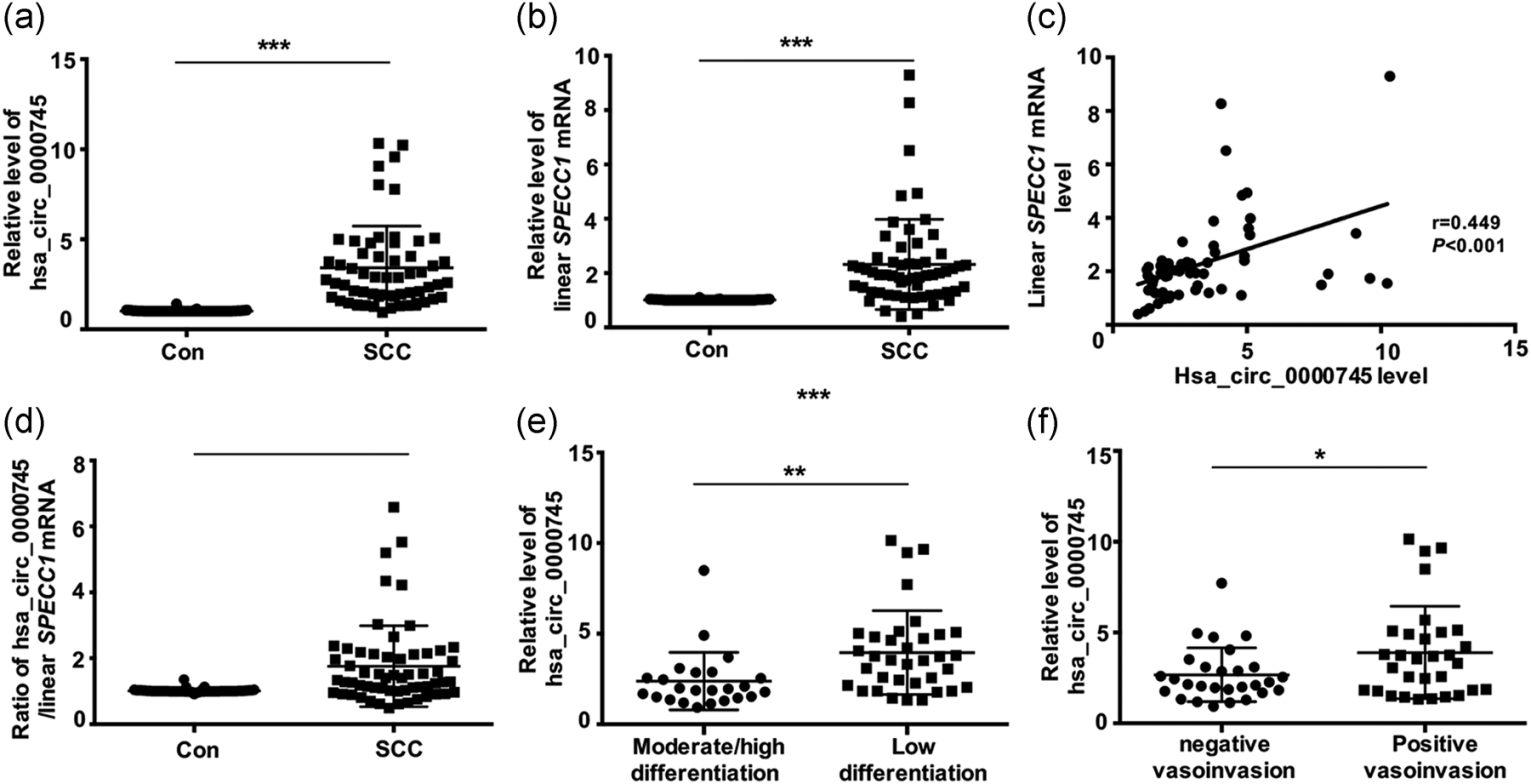
Expression levels and correlation analysis of hsa_circ_0000745 and linear *SPECC1* mRNA in SCC. (a) The expression level of hsa_circ_0000745 in SCC was higher than that in normal cervical tissue (Con). (b) The expression level of linear *SPECC1* mRNA in SCC was significantly higher than that in Con. (c) The level of hsa_circ_0000745 in SCC was positively correlated with the level of linear *SPECC1* mRNA. (d) The ratio of hsa_circ_0000745/linear *SPECC1* mRNA was higher in SCC than in Con. (e) The expression of hsa_circ_0000745 in patients with low differentiation was higher than that in patients with high or moderate differentiation. (f) The expression of hsa_circ_0000745 in patients with positive vascular/lymphatic invasion was higher than that in patients with negative vascular/lymphatic invasion. *p < .05, **p < .01, *** *p* < .001. mRNA, messenger RNA; SCC, squamous cell carcinoma

In the circRNA microarray, circ‐0745 was detected in cervical cells using a probe specific for the junction site of circ‐0745. circ‐0745 displayed significant differences in expression between SCC and normal cervical cells (Figure 1c). We detected the levels of both circ‐0745 and the linear *SPECC1* mRNA in 59 paired SCC and paracancerous cervical tissues using qRT‐PCR. Compared with the control tissues, circ‐0745 was upregulated in SCC (*p* < .001; Figure 2a), consistent with the result from the circRNA microarray. The linear *SPECC1* mRNA was also upregulated in the SCC group (*p* < .001; Figure 2b). Furthermore, circ‐0745 positively correlated with the expression of the linear *SPECC1* mRNA in SCC tissues (*r *= 0.449, *p* < .001; Figure 2c). Interestingly, a higher circ‐0745/linear *SPECC1* mRNA ratio was observed in SCC tissues than in the control (*p* < .001; Figure 2d). Based on these findings, circ‐0745 was involved in the development of SCC.

### 3.3 circ‐0745 is related to patients’ clinicopathological parameters

The relationships between the levels of circ‐0745 and several clinicopathological characteristics (Table 3) that may affect the treatment and prognosis of patients with CC were analyzed. Compared with patients with SCC presenting with well or moderately differentiated tumors, patients with poorly differentiated tumors exhibited substantially increased circ‐0745 expression (*p* = .005; Figure 2e). Patients with positive vascular/lymphatic invasion exhibited higher levels of circ‐0745 than patients without vascular/lymphatic invasion (*p* = .026; Figure 2f). No significant correlation was detected between circ‐0745 expression and other prognostic factors in patients with SCC, including the clinical stage, tumor size, and lymph node metastasis. Poor differentiation and positive vascular/lymphatic invasion often indicate an unfavorable prognosis and follow‐up treatment with chemoradiotherapy. The observed associations suggested that patients with SCC presenting with higher levels of circ‐0745 may have a worse prognosis than patients with lower levels of circ‐0745.

**Table 3:** Relationships between the hsa_circ_0000745 expression level in SCC and the clinicopathologic parameters of patients

Characteristics	No. of patients	Mean ± *SD*	*p* Value
FIGO stage			
I	48	3.296 ± 2.145	.873
II	11	3.414 ± 2.429	
Tumor differentiation			
Moderate/high	24	2.381 ± 1.588	.005^**^
Low	35	3.960 ± 2.313	
Tumor size (cm)			
< 4	43	3.356 ± 2.311	.825
≥ 4	16	3.213 ± 1.840	
Vascular/lymphatic invasion			
Negative	28	2.673 ± 1.482	.026^*^
Positive	31	3.900 ± 2.543	
Lymph node metastases			
Negative	43	3.583 ± 2.373	.126
Positive	16	2.604 ± 1.361	

Abbreviations: FIGO, International Federation of Gynecology and Obstetrics; SCC, squamous cell carcinoma.

^*^ *p* < .05.

^**^ *p* < .01.

In this study, we collected specimens from only CC patients with Stage I and II because patients with more advanced stages were treated with radiotherapy or chemotherapy rather than with surgery. Because we were unable to collect a sufficient number of cancer tissues from individuals with Stage III or Stage IV disease, the analysis between circ‐0745 expression and the patients’ clinical stages was restricted to early stages.

### 3.4 Inhibition of circ‐0745 suppresses CC cell proliferation

**Figure 3:**
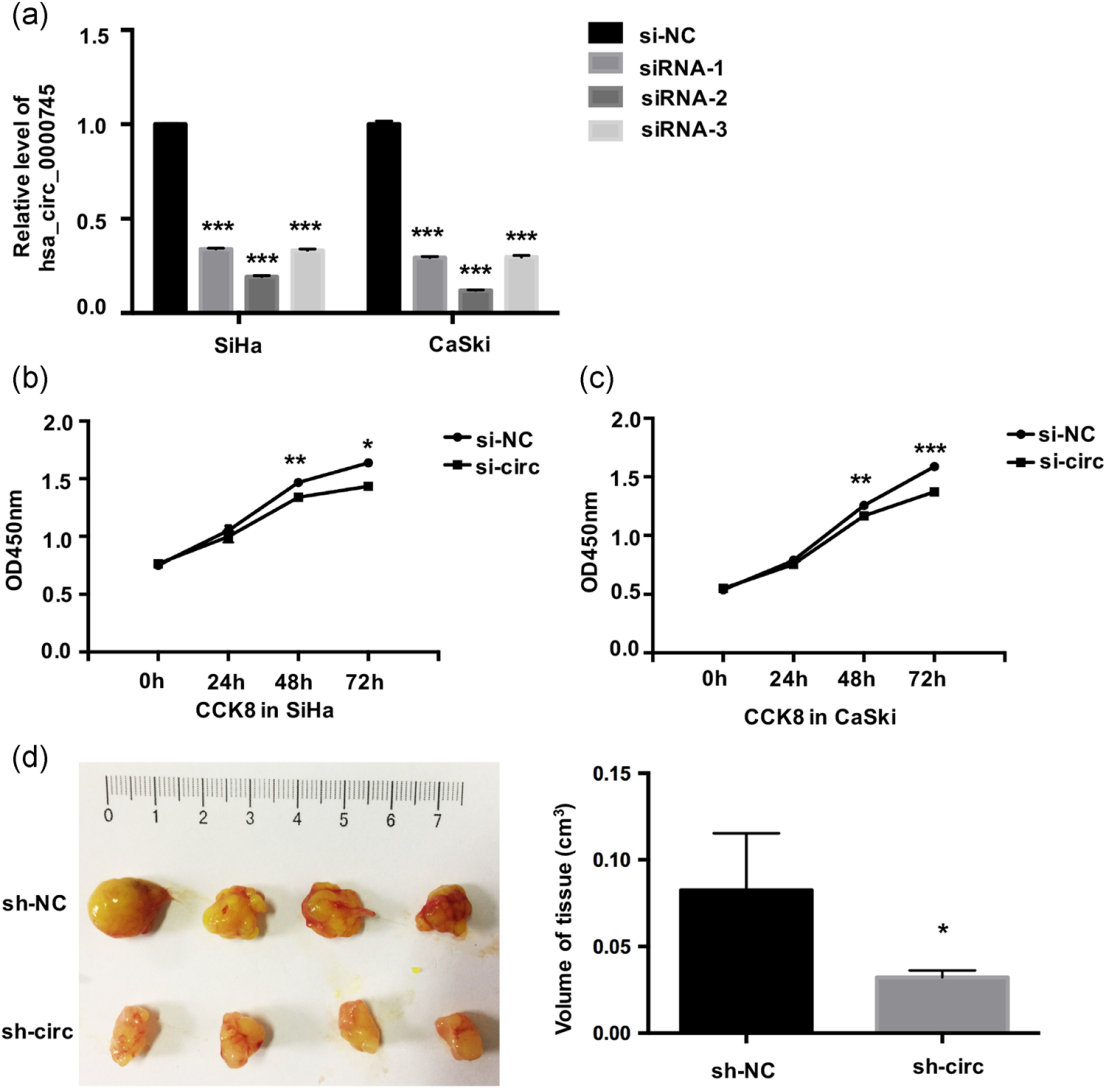
Knockdown of hsa_circ_0000745 inhibits CC cell proliferation. (a) The expression levels of hsa_circ_0000745 in SiHa and CaSki cells were determined by qRT‐PCR after transfection with three hsa_circ_0000745 siRNAs individually (siRNA‐1, siRNA‐2 and siRNA‐3). (b) The CCK‐8 assay showed that hsa_circ_0000745 knockdown (si‐circ) obviously suppressed the proliferation of SiHa cells compared with cells transfected with the control (si‐NC). (c) The CCK‐8 assay showed that hsa_circ_0000745 knockdown (si‐circ) obviously suppressed the proliferation of CaSki cells compared with cells transfected with si‐NC. (d) The subcutaneous tumor volume of BALB/c mice in the sh‐circ‐0000745 group was smaller than that in the control (sh‐NC). *p < .05, **p < .01, ***p < .001. CC, cervical cancer; CCK‐8, Cell Counting Kit‐8; qRT‐PCR, quantitative reverse transcription polymerase chain reaction; si‐NC, negative siRNA control; siRNA, small interfering RNA [Color figure can be viewed at wileyonlinelibrary.com]

In SiHa and CaSki cells, specifically designed siRNAs were used to knockdown circ‐0745 expression. circ‐0745 expression was significantly reduced in cells transfected with siRNA‐2; this construct was used in all subsequent analyses (Figure 3a). Compared with the negative‐control treatment, circ‐0745 knockdown obviously suppressed the proliferation ability of SiHa and CaSki cells, as examined using CCK‐8 assays (Figure 3b,c). Flow cytometry was conducted to detect cell apoptosis after circ‐0745 expression was silenced. Changes in the expression of circ‐0745 had no effect on the apoptosis of SiHa or CaSki cells (*p* > .05).

The role of circ‐0745 in promoting CC cell proliferation was tested by generating a subcutaneous tumor model in nude mice. Tumor volume = Length × Width × Height × *π*/6. The volume of neoplasms in the sh‐circ_0000745 group was markedly smaller than in the control (*p* < .05; Figure 3d), which indicated that inhibiting hsa_circ_0000745 obviously suppressed CC cells’ proliferation in vivo.

### 3.5 Inhibition of circ‐0745 inhibits CC cells migration and invasion by upregulating E‐CAD

**Figure 4:**
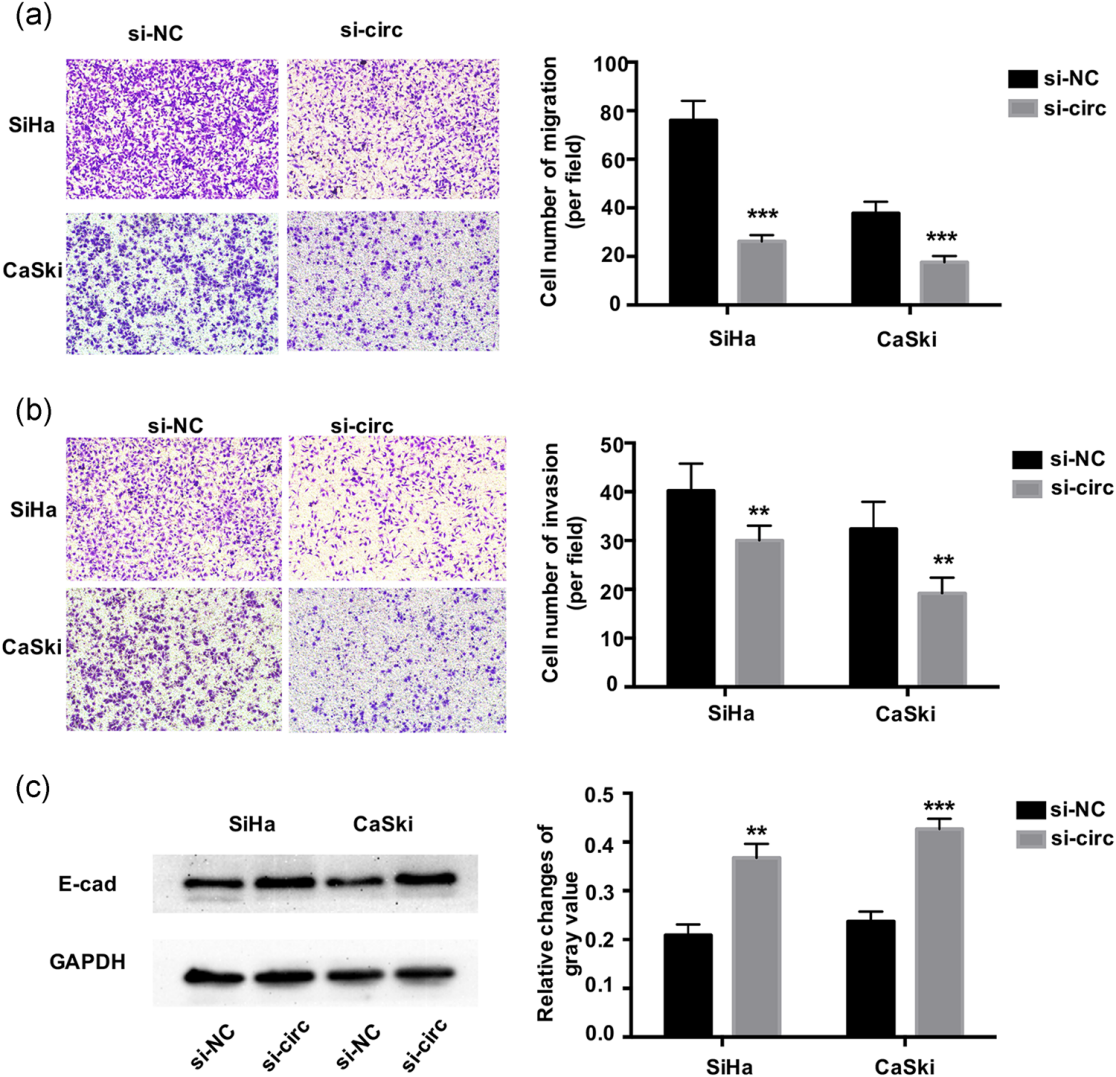
Knockdown of hsa_circ_0000745 inhibits CC cell migration and invasion. (a) Transwell assays showed knockdown of hsa_circ_0000745 (si‐circ) obviously suppressed cell migration in SiHa and CaSki cells compared with si‐NC. (b) Transwell assays showed that knockdown of hsa_circ_0000745 (si‐circ) obviously suppressed cell invasion in SiHa and CaSki cells compared with si‐NC. (c) Knockdown of hsa_circ_0000745 led to elevated expression of E‐cad in SiHa and CaSki cells. *p < .05, **p < .01, ***p < .001. CC, cervical cancer; si‐NC, negative siRNA control [Color figure can be viewed at wileyonlinelibrary.com]

SiHa and CasKi cells showed decreased migration through a Transwell chamber after downregulation of circ‐0745 (*p* < .001; Figure 4a), there was a similar trend detected in the invasion experiments (*p* < .01; Figure 4b). Based on these results, knockdown of circ‐0745 noticeably inhibited the migratory and invasive ability of SiHa and CaSki cells. We also measured the relationship between circ‐0745 and E‐cad expression. After downregulating the expression of circ‐0745, the levels of E‐cad were increased in both SiHa and CaSki cells (*p*_SiHa_ < .01 and *p*_CaSki_ < .001; Figure 4c), suggesting that the downregulation of circ‐0745 induced E‐cad expression to suppress CC cells migration and invasion.

## 4 DISCUSSION

Although previously neglected as byproducts, circRNAs first attracted attention after the identification of ciRS‐7, a circRNA also known as CDR1as. ciRS‐7, a designated sponge and inhibitor of miR‐7, conceptually changed the understanding of the mechanisms of miRNAs and gene regulation networks (Hansen, Jensen, et al., 2013; Hansen, Kjems, & Damgaard, 2013). Due to the wide use of high‐throughput sequencing and microarray assays, increasing numbers of circRNAs have been identified in human samples. Currently, because of ongoing research and the publication of new information about circRNA biogenesis, regulation, and expression patterns, functions of circRNAs in addition to their miRNA‐sponge activities have been identified, including as competitors with linear mRNAs (Ashwal‐Fluss et al., 2014), protein decoys (Abdelmohsen et al., 2017; Du et al., 2017), and protein translation (Liang et al., 2019; Yang et al., 2017). Notably, circRNAs have attracted increasing attention from cancer researchers (Bach, Lee, & Sood, 2019; Hsiao et al., 2017; Zhang & Xin, 2018). Understanding the roles of circRNAs will provide new insights into oncogenesis and tumor development, thereby providing a foundation for novel cancer detection and treatment methods. Here, we identified differentially expressed circRNAs in tumor tissues from CC patients compared with matched adjacent cervical tissues. One hundred seventy‐eight aberrantly expressed circRNAs were detected and subjected to a preliminary analysis. As differentially expressed circRNAs have different functions in carcinoma genesis and progression, the specific roles of dysregulated circRNAs must be studied.

We further investigated one differentially expressed circRNA, circ‐0745, and identified an important role for this circRNA in CC development. circ‐0745 is derived from the gene *SPECC1*. After transcription, the pre‐mRNA of *SPECC1* is spliced into the linear *SPECC1* mRNA and the circRNA isoform, circ‐0745. The linear isoform functions in cancer development, participating in cell proliferation and apoptosis (D’Agostino & Giordano, 2008); the function of circ‐0745 in the development of malignant tumors attracted our interest. circ‐0745 is reduced in GC patients’ tumor tissues and blood plasma. In addition, circ‐0745 correlates with the tumor‐node‐metastasis stage, revealing circ‐0745 plays a pivotal role and is a promising marker in GC (Huang, He, Liang, Huang, & Zhu, 2017). In our study, circ‐0745 was aberrantly upregulated in CC tissues, according to both circRNA microarray and qRT‐PCR results. These results suggest the same circRNA may be differentially expressed in different tissues or organs and play different roles in different diseases.

Correlation analysis between circ‐0745 expression and the clinical features of CC patients showed that patients with poor differentiation or positive vascular/lymphatic invasion exhibited much higher levels of circ‐0745 than patients with well or moderately differentiated tumors or no vascular/lymphatic invasion. Patients with poor tumor differentiation and/or positive vascular/lymphatic invasion have a worse prognosis and often need postoperative radiotherapy or chemotherapy to reduce the risk of recurrence. Thus, circ‐0745 is important in the progression of CC and may influence the prognosis of patients with CC.

Some circRNAs work as tumor suppressors or oncogenes in different types of malignant tumors. For example, circRNA‐000284 is an oncogene and promotes the progression of CC via the miR‐506/Snail‐2 pathway (Ma, Yao, Yu, Chen, & Li, 2018), whereas circ‐ITCH is a tumor suppressor by suppressing the activity of miR‐17/miR‐224 and upregulating their target genes in bladder carcinoma (Yang et al., 2018). We downregulated circ‐0745 in cancer cells to investigate its function in CC. The CC cells with a low level of circ‐0745 exhibited reduced proliferation in vitro and in vivo and reduced invasion and migration in vitro. Thus, circ‐0745 functions as an oncogene by enhancing the malignancy of CC cells, consistent with the finding that patients with poor tumor differentiation or positive vascular/lymphatic invasion presented much higher expression of circ‐0745 than patients with well or moderately differentiated tumors and no invasion. Based on these findings, the inhibition of circ‐0745 represents a direction for exploring new therapies for CC.

In epithelial cells, E‐cad is essential for maintaining the homeostasis of the polarized epithelial monolayer. Downregulation of E‐cad is common in tumors manifesting from epithelial tissues and promotes cell migration and invasion (Peng et al., 2018; Sarrio et al., 2004). The downregulation of E‐cad during cancer progression is generally considered the result of the epithelial‐to‐mesenchymal transition (EMT). Reduced E‐cad expression during EMT is often considered to be a major driver of cancer progression and metastasis (Kourtidis, Lu, Pence, & Anastasiadis, 2017).

As metastasis is considered the primary cause of death in tumor patients, the mechanism by which circ‐0745 promotes CC cell metastasis is worth exploring. Inhibition of circ‐0745 elevated the expression of E‐cad in CC cells, resulting in the suppression of cell invasion and migration. Based on these results, increased circ‐0745 expression inhibits E‐cad expression in CC cells, subsequently promoting the metastasis of CC cells and resulting in cancer progression.

In summary, we highlight the expression of circRNAs in human CC. circ‐0745 is confirmed to be overexpressed in CC and may be related to the prognosis of patients with CC. Functionally and mechanistically, circ‐0745 promotes the cellular ability to proliferate, migrate and invade by reducing the expression of E‐cad, indicating its role as a tumor promoter in CC progression. Based on our data, circ‐0745 plays an important part in promoting CC development and represents a candidate marker of patient prognosis and a target for clinical treatments for CC.

## CONFLICT OF INTERESTS

The authors declare that there are no conflict of interests.

## AUTHOR CONTRIBUTIONS

J.J. performed the experiments and wrote the manuscript. T.Z. and X.J. participated in the experimental work and collection of data. T.H. and L.Z. analyzed the data. D.M. participated in the design and coordination of experimental work. B.C. designed the research, provided reagents and revised the paper. All authors read and approved the final manuscript.

## DATA ACCESSIBILITY

The circRNA microarray data of this study are openly available in the NCBI Gene Expression Omnibus (GEO) DataSets (https://www.ncbi.nlm.nih.gov/geo/), accession number GSE102686.

## References

[jcp29045-bib-0001] Abdelmohsen, K. , Panda, A. C. , Munk, R. , Grammatikakis, I. , Dudekula, D. B. , De, S. , & Gorospe, M. (2017). Identification of HuR target circular RNAs uncovers suppression of PABPN1 translation by circPABPN1. RNA Biology, 14(3), 361–369. 28080204 10.1080/15476286.2017.1279788 PMC5367248

[jcp29045-bib-0002] Ashwal‐Fluss, R. , Meyer, M. , Pamudurti, N. R. , Ivanov, A. , Bartok, O. , Hanan, M. , & Kadener, S. (2014). circRNA biogenesis competes with pre‐mRNA splicing. Molecular Cell, 56(1), 55–66. 25242144 10.1016/j.molcel.2014.08.019

[jcp29045-bib-0003] Bach, D. H. , Lee, S. K. , & Sood, A. K. (2019). Circular RNAs in cancer. Molecular Therapy. Nucleic Acids, 16, 118–129. 30861414 10.1016/j.omtn.2019.02.005 PMC6411617

[jcp29045-bib-0004] Cai, X. , Zhao, Z. , Dong, J. , Lv, Q. , Yun, B. , Liu, J. , & Li, J. (2019). Circular RNA circBACH2 plays a role in papillary thyroid carcinoma by sponging miR‐139‐5p and regulating LMO4 expression. Cell Death & Disease, 10(3), 184. 30796202 10.1038/s41419-019-1439-y PMC6385235

[jcp29045-bib-0005] D’Agostino, L. , & Giordano, A. (2008). Possible functional role of NSPs in cancer. Cell Cycle, 7(12), 1810–1827. 18763323 10.4161/cc.7.12.6023

[jcp29045-bib-0006] Du, W. W. , Yang, W. , Chen, Y. , Wu, Z. K. , Foster, F. S. , Yang, Z. , & Yang, B. B. (2017). Foxo3 circular RNA promotes cardiac senescence by modulating multiple factors associated with stress and senescence responses. European Heart Journal, 38(18), 1402–1412. 26873092 10.1093/eurheartj/ehw001

[jcp29045-bib-0007] Ferlay, J. , Soerjomataram, I. , Dikshit, R. , Eser, S. , Mathers, C. , Rebelo, M. , & Bray, F. (2015). Cancer incidence and mortality worldwide: Sources, methods and major patterns in GLOBOCAN 2012. International Journal of Cancer, 136(5), E359–E386. 25220842 10.1002/ijc.29210

[jcp29045-bib-0008] Ginsburg, O. , Bray, F. , Coleman, M. P. , Vanderpuye, V. , Eniu, A. , Kotha, S. R. , & Conteh, L. (2017). The global burden of women's cancers: A grand challenge in global health. Lancet, 389(10071), 847–860. 27814965 10.1016/S0140-6736(16)31392-7 PMC6191029

[jcp29045-bib-0009] Han, D. , Li, J. , Wang, H. , Su, X. , Hou, J. , Gu, Y. , & Cao, X. (2017). Circular RNA circMTO1 acts as the sponge of microRNA‐9 to suppress hepatocellular carcinoma progression. Hepatology, 66(4), 1151–1164. 28520103 10.1002/hep.29270

[jcp29045-bib-0010] Hansen, T. B. , Jensen, T. I. , Clausen, B. H. , Bramsen, J. B. , Finsen, B. , Damgaard, C. K. , & Kjems, J. (2013). Natural RNA circles function as efficient microRNA sponges. Nature, 495(7441), 384–388. 23446346 10.1038/nature11993

[jcp29045-bib-0011] Hansen, T. B. , Kjems, J. , & Damgaard, C. K. (2013). Circular RNA and miR‐7 in cancer. Cancer Research, 73(18), 5609–5612. 24014594 10.1158/0008-5472.CAN-13-1568

[jcp29045-bib-0012] Hsiao, K. Y. , Lin, Y. C. , Gupta, S. K. , Chang, N. , Yen, L. , Sun, H. S. , & Tsai, S. J. (2017). Noncoding effects of circular RNA CCDC66 promote colon cancer growth and metastasis. Cancer Research, 77(9), 2339–2350. 28249903 10.1158/0008-5472.CAN-16-1883 PMC5910173

[jcp29045-bib-0013] Hu, C. , Wang, Y. , Li, A. , Zhang, J. , Xue, F. , & Zhu, L. (2019). Overexpressed circ_0067934 acts as an oncogene to facilitate cervical cancer progression via the miR‐545/EIF3C axis. Journal of Cellular Physiology, 234(6), 9225–9232. 30362562 10.1002/jcp.27601

[jcp29045-bib-0014] Huang, M. , He, Y. R. , Liang, L. C. , Huang, Q. , & Zhu, Z. Q. (2017). Circular RNA hsa_circ_0000745 may serve as a diagnostic marker for gastric cancer. World Journal of Gastroenterology, 23(34), 6330–6338. 28974900 10.3748/wjg.v23.i34.6330 PMC5603500

[jcp29045-bib-0015] Kourtidis, A. , Lu, R. , Pence, L. J. , & Anastasiadis, P. Z. (2017). A central role for cadherin signaling in cancer. Experimental Cell Research, 358(1), 78–85. 28412244 10.1016/j.yexcr.2017.04.006 PMC5544584

[jcp29045-bib-0016] Li, J. Q. , Yang, J. , Zhou, P. , Le, Y. P. , Zhou, C. W. , Wang, S. M. , & Gong, Z. H. (2015). Circular RNAs in cancer: Novel insights into origins, properties, functions and implications. American Journal of Cancer Research, 5(2), 472–480. 25973291 PMC4396047

[jcp29045-bib-0017] Liang, W.‐C. , Wong, C.‐W. , Liang, P.‐P. , Shi, M. , Cao, Y. , Rao, S.‐T. , & Zhang, J.‐F. (2019). Translation of the circular RNA circβ‐catenin promotes liver cancer cell growth through activation of the Wnt pathway. Genome Biology, 20(1), 84. 31027518 10.1186/s13059-019-1685-4 PMC6486691

[jcp29045-bib-0018] Ma, H. B. , Yao, Y. N. , Yu, J. J. , Chen, X. X. , & Li, H. F. (2018). Extensive profiling of circular RNAs and the potential regulatory role of circRNA‐000284 in cell proliferation and invasion of cervical cancer via sponging miR‐506. American Journal of Translational Research, 10(2), 592–604. 29511454 PMC5835825

[jcp29045-bib-0019] Meng, X. , Li, X. , Zhang, P. , Wang, J. , Zhou, Y. , & Chen, M. (2017). Circular RNA: An emerging key player in RNA world. Briefings in Bioinformatics, 18(4), 547–557. 27255916 10.1093/bib/bbw045

[jcp29045-bib-0020] Munoz, N. , Castellsague, X. , de Gonzalez, A. B. , & Gissmann, L. (2006). Chapter 1: HPV in the etiology of human cancer. Vaccine, 24(Suppl. 3), S3/1–S3/10. 10.1016/j.vaccine.2006.05.115 16949995

[jcp29045-bib-0021] Peng, S. Y. , Tu, H. F. , Yang, C. C. , Wu, C. H. , Liu, C. J. , Chang, K. W. , & Lin, S. C. (2018). miR‐134 targets PDCD7 to reduce E‐cadherin expression and enhance oral cancer progression. International Journal of Cancer, 143(11), 2892–2904. 29971778 10.1002/ijc.31638

[jcp29045-bib-0022] Rong, D. , Lu, C. , Zhang, B. , Fu, K. , Zhao, S. , Tang, W. , & Cao, H. (2019). CircPSMC3 suppresses the proliferation and metastasis of gastric cancer by acting as a competitive endogenous RNA through sponging miR‐296‐5p. Molecular Cancer, 18(1), 25. 30777076 10.1186/s12943-019-0958-6 PMC6378730

[jcp29045-bib-0023] Sarrio, D. , Perez‐Mies, B. , Hardisson, D. , Moreno‐Bueno, G. , Suarez, A. , Cano, A. , & Palacios, J. (2004). Cytoplasmic localization of p120ctn and E‐cadherin loss characterize lobular breast carcinoma from preinvasive to metastatic lesions. Oncogene, 23(19), 3272–3283. 15077190 10.1038/sj.onc.1207439

[jcp29045-bib-0024] Suzuki, H. , & Tsukahara, T. (2014). A view of pre‐mRNA splicing from RNase R resistant RNAs. International Journal of Molecular Sciences, 15(6), 9331–9342. 24865493 10.3390/ijms15069331 PMC4100097

[jcp29045-bib-0025] Wang, F. , Nazarali, A. J. , & Ji, S. (2016). Circular RNAs as potential biomarkers for cancer diagnosis and therapy. American Journal of Cancer Research, 6(6), 1167–1176. 27429839 PMC4937728

[jcp29045-bib-0026] Wang, H. , Zhao, Y. , Chen, M. , & Cui, J. (2017). Identification of novel long non‐coding and circular RNAs in human papillomavirus‐mediated cervical cancer. Frontiers in Microbiology, 8, 1720. 28970820 10.3389/fmicb.2017.01720 PMC5609541

[jcp29045-bib-0027] Wilusz, J. E. , & Sharp, P. A. (2013). Molecular biology. A circuitous route to noncoding RNA. Science, 340(6131), 440–441. 23620042 10.1126/science.1238522 PMC4063205

[jcp29045-bib-0028] Yang, C. , Yuan, W. , Yang, X. , Li, P. , Wang, J. , Han, J. , & Zhang, W. (2018). Circular RNA circ‐ITCH inhibits bladder cancer progression by sponging miR‐17/miR‐224 and regulating p21, PTEN expression. Molecular Cancer, 17(1), 19. 29386015 10.1186/s12943-018-0771-7 PMC5793418

[jcp29045-bib-0029] Yang, Y. , Fan, X. , Mao, M. , Song, X. , Wu, P. , Zhang, Y. , & Wang, Z. (2017). Extensive translation of circular RNAs driven by N(6)‐methyladenosine. Cell Research, 27(5), 626–641. 28281539 10.1038/cr.2017.31 PMC5520850

[jcp29045-bib-0030] Yao, Z. , Luo, J. , Hu, K. , Lin, J. , Huang, H. , Wang, Q. , & Yang, Y. (2017). ZKSCAN1 gene and its related circular RNA (circZKSCAN1) both inhibit hepatocellular carcinoma cell growth, migration, and invasion but through different signaling pathways. Molecular Oncology, 11(4), 422–437. 28211215 10.1002/1878-0261.12045 PMC5527481

[jcp29045-bib-0031] Yu, D. , Li, Y. , Ming, Z. , Wang, H. , Dong, Z. , Qiu, L. , & Wang, T. (2018). Comprehensive circular RNA expression profile in radiation‐treated HeLa cells and analysis of radioresistance‐related circRNAs. Peer Journal, 6, e5011. 10.7717/peerj.5011 PMC6005163 29922514

[jcp29045-bib-0032] Zhang, M. , & Xin, Y. (2018). Circular RNAs: A new frontier for cancer diagnosis and therapy. Journal of Hematology & Oncology, 11(1), 21. 29433541 10.1186/s13045-018-0569-5 PMC5809913

